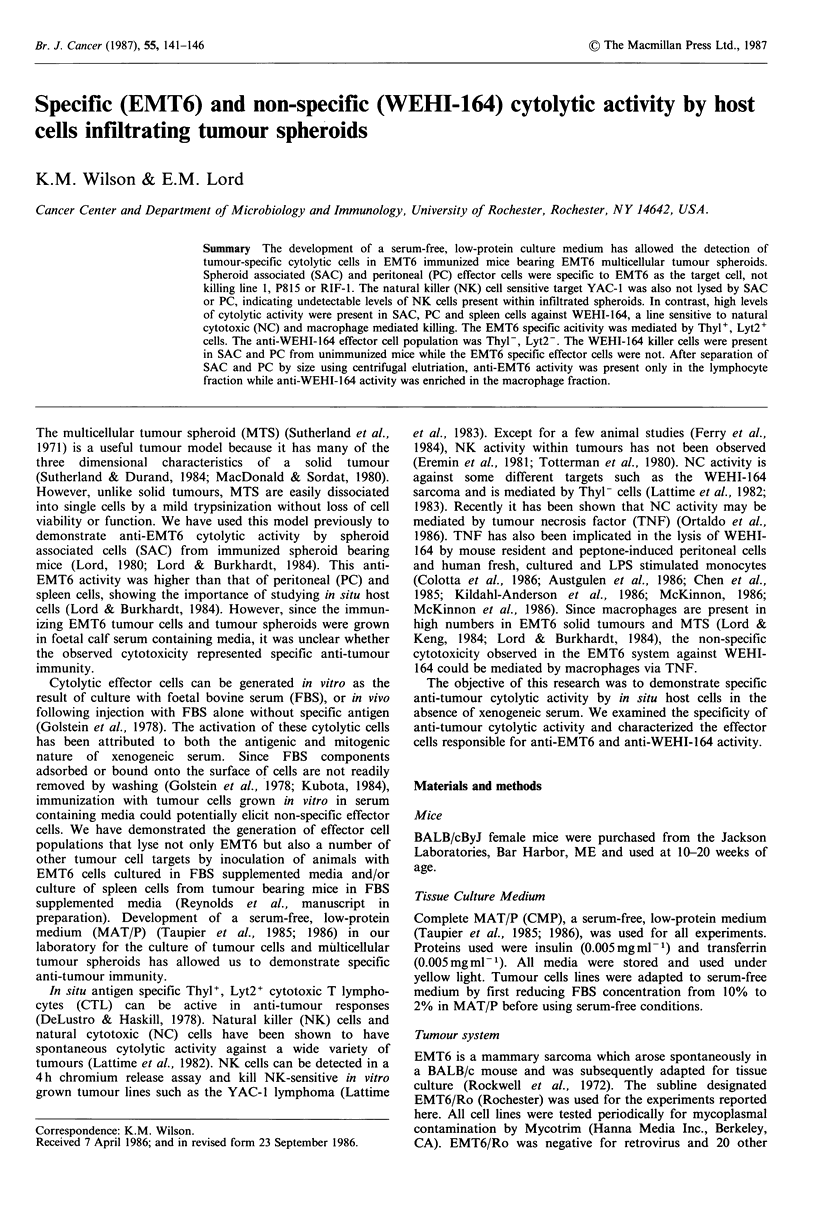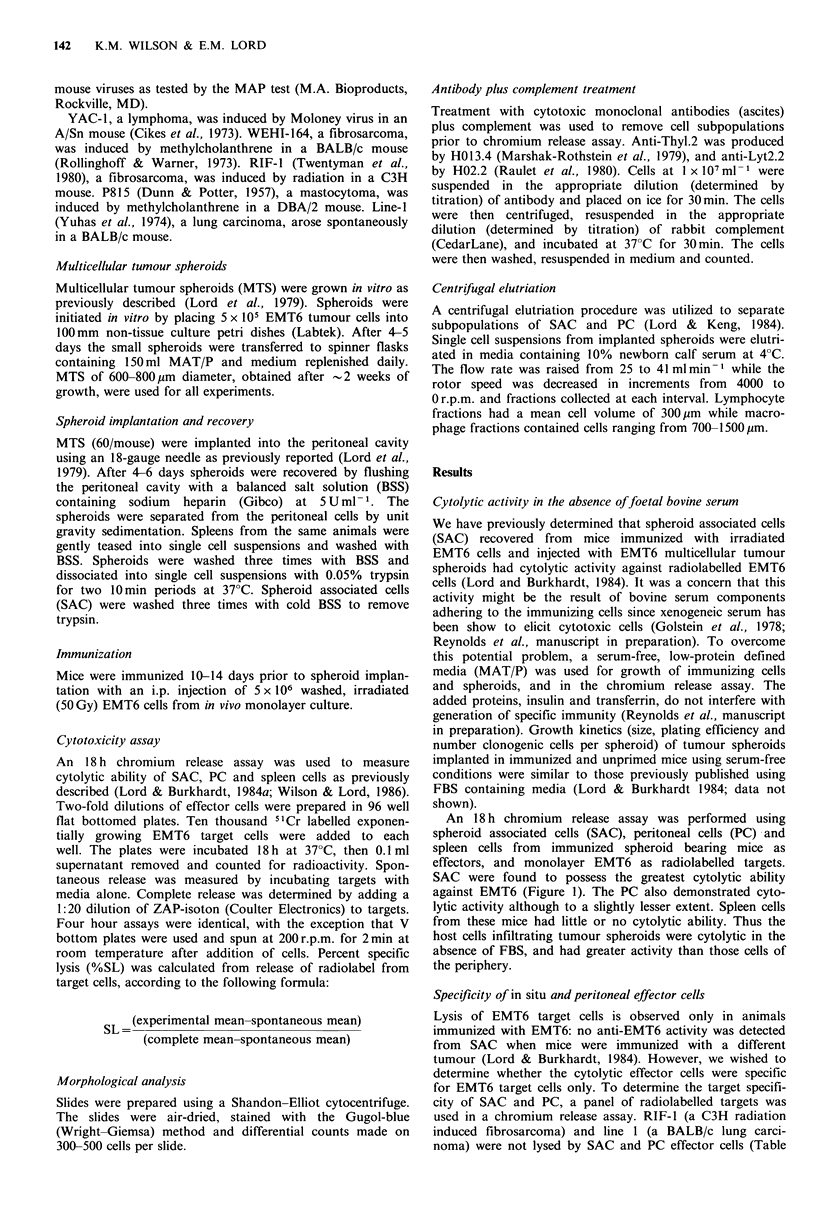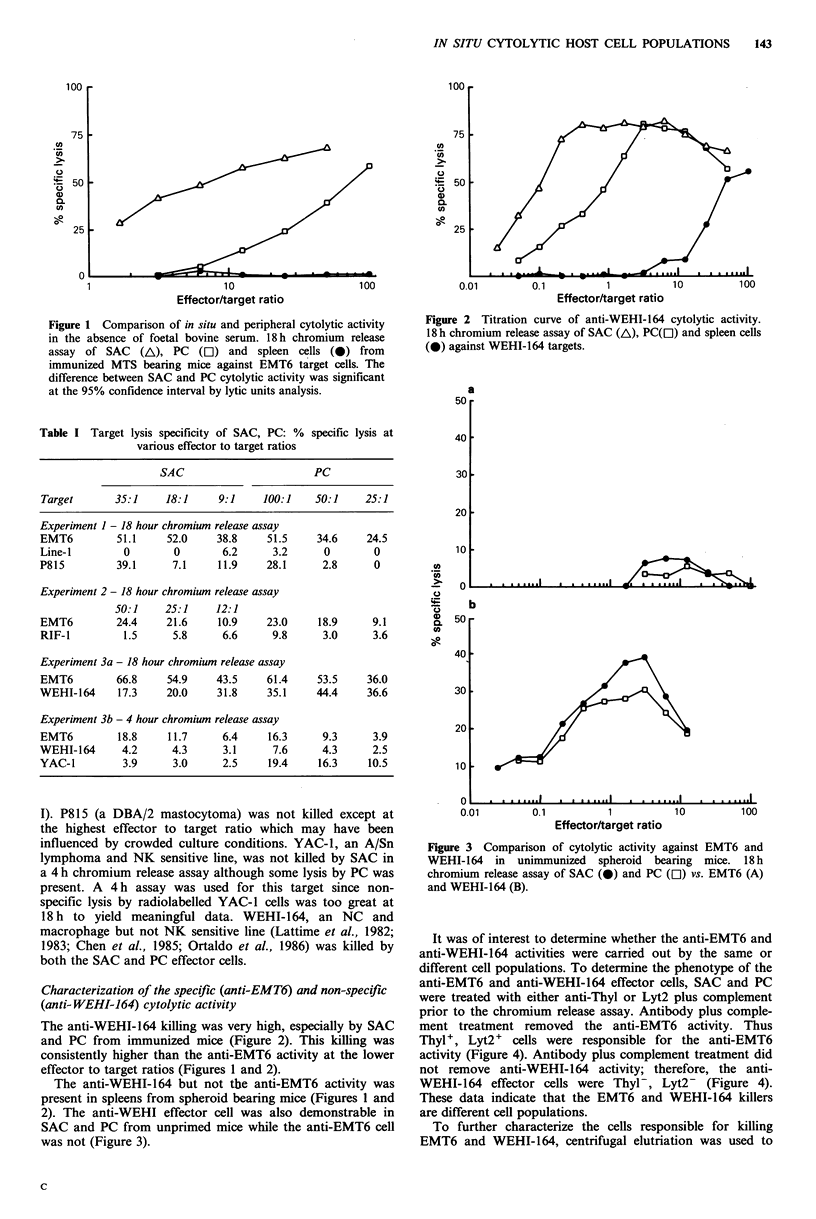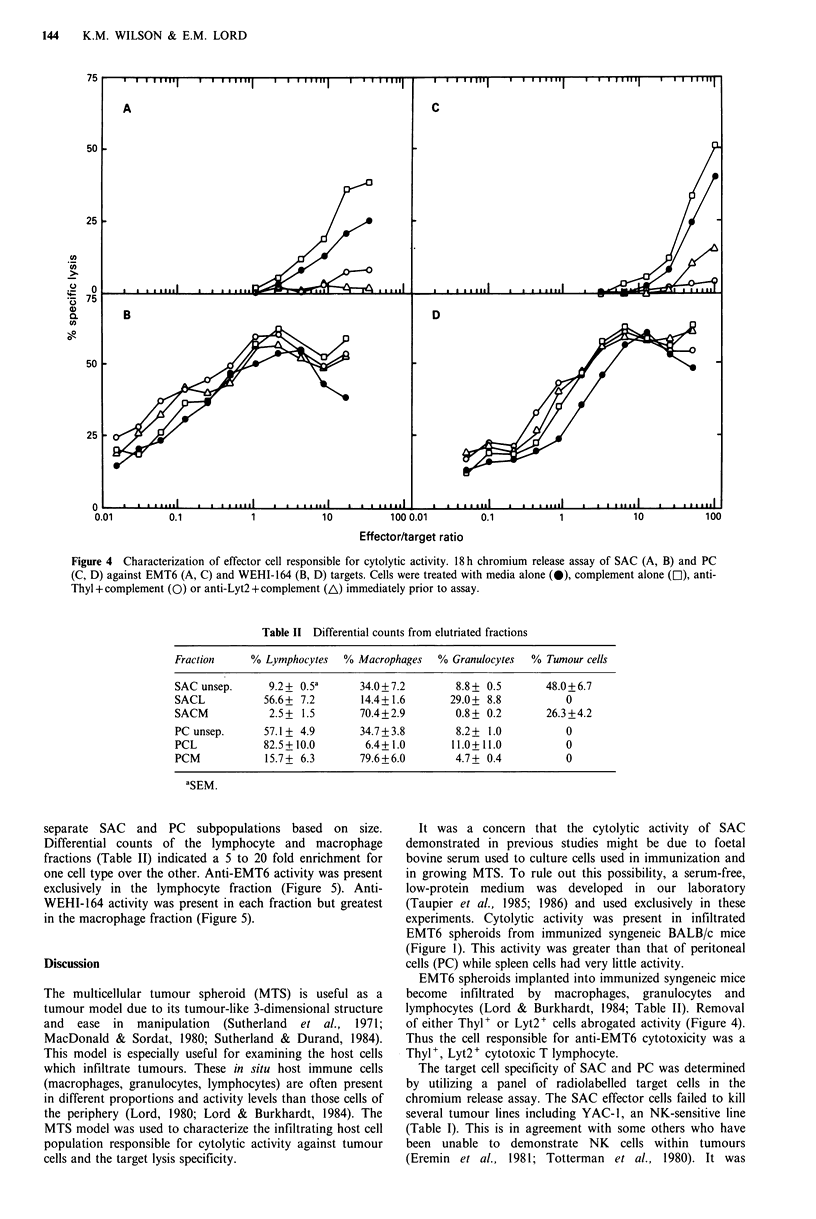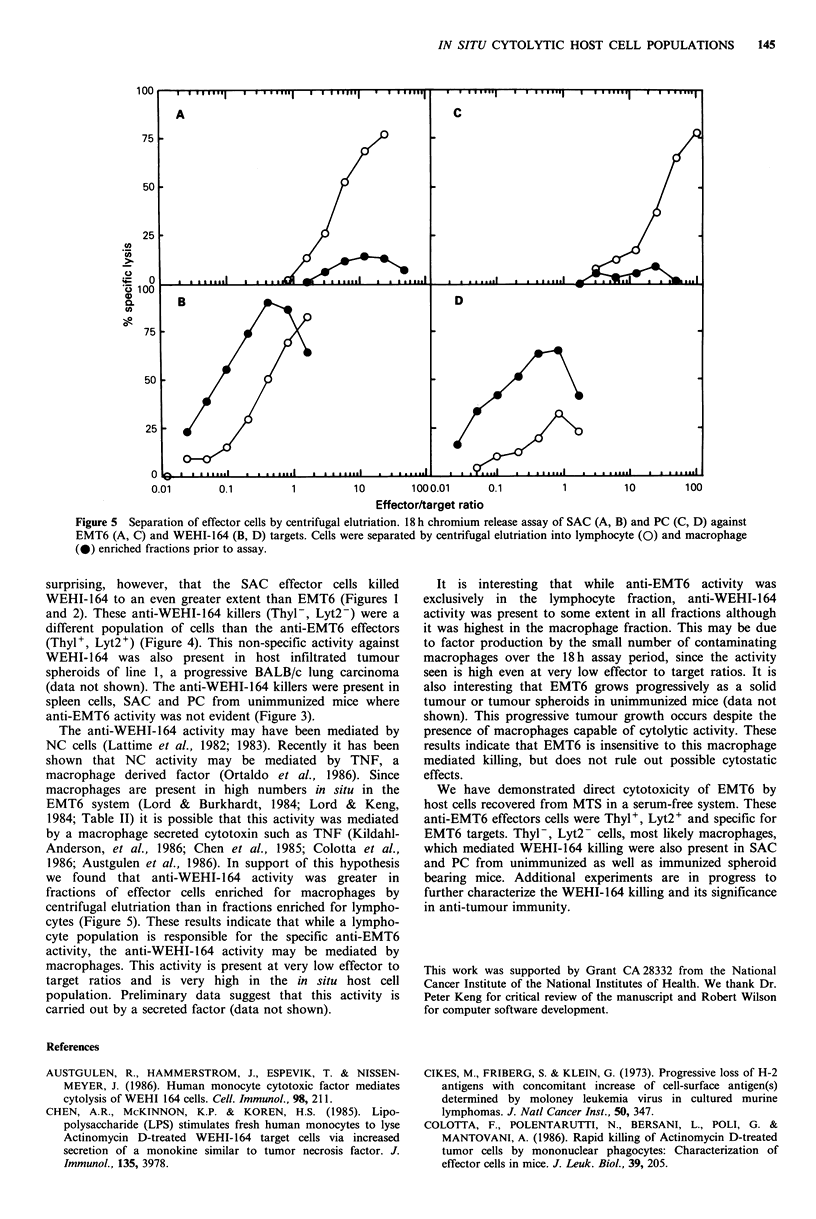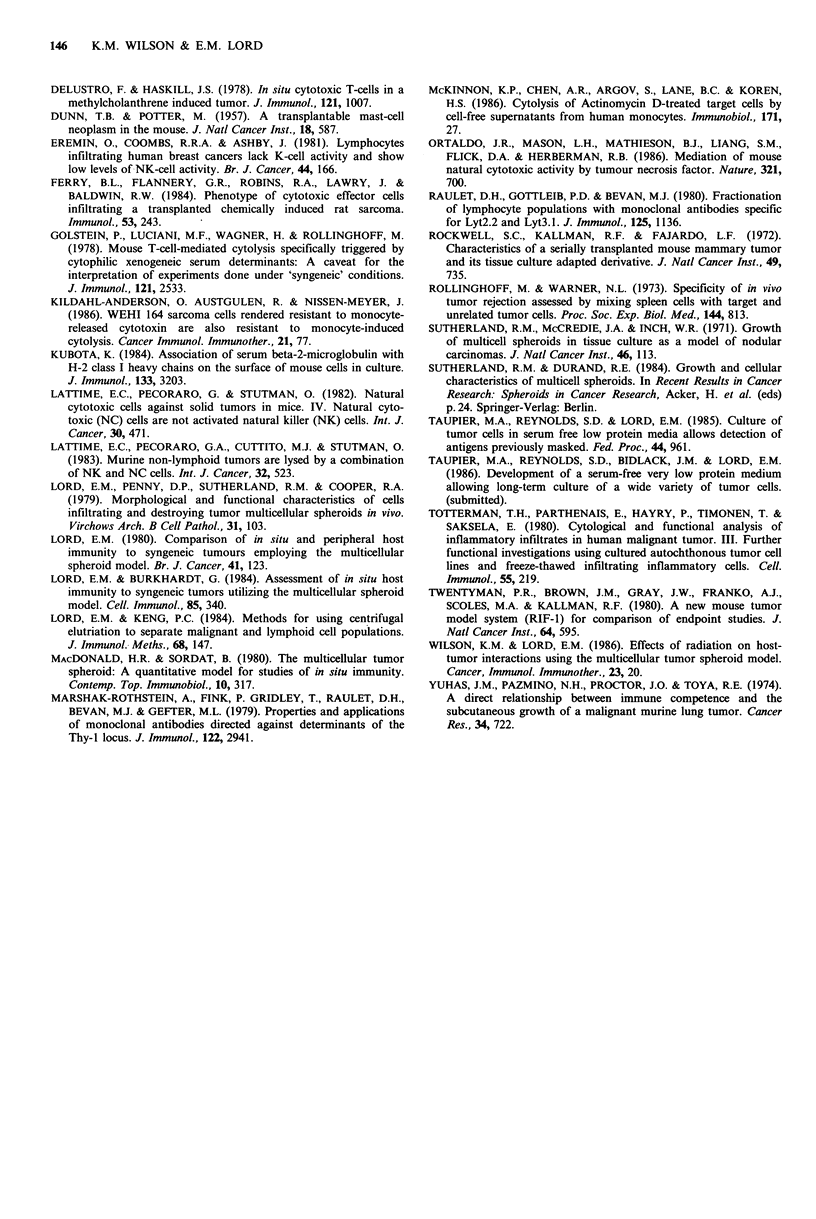# Specific (EMT6) and non-specific (WEHI-164) cytolytic activity by host cells infiltrating tumour spheroids.

**DOI:** 10.1038/bjc.1987.29

**Published:** 1987-02

**Authors:** K. M. Wilson, E. M. Lord

## Abstract

The development of a serum-free, low-protein culture medium has allowed the detection of tumour-specific cytolytic cells in EMT6 immunized mice bearing EMT6 multicellular tumour spheroids. Spheroid associated (SAC) and peritoneal (PC) effector cells were specific to EMT6 as the target cell, not killing line 1, P815 or RIF-1. The natural killer (NK) cell sensitive target YAC-1 was also not lysed by SAC or PC, indicating undetectable levels of NK cells present within infiltrated spheroids. In contrast, high levels of cytolytic activity were present in SAC, PC and spleen cells against WEHI-164, a line sensitive to natural cytotoxic (NC) and macrophage mediated killing. The EMT6 specific activity was mediated by Thyl+, Lyt2+ cells. The anti-WEHI-164 effector cell population was Thyl-, Lyt2-. The WEHI-164 killer cells were present in SAC and PC from unimmunized mice while the EMT6 specific effector cells were not. After separation of SAC and PC by size using centrifugal elutriation, anti-EMT6 activity was present only in the lymphocyte fraction while anti-WEHI-164 activity was enriched in the macrophage fraction.


*Br. J. Cancer* (1987), **55**, 141–146 © The Macmillan Press Ltd., 1987

### Specific (EMT6) and non-specific (WEHI-164) cytolytic activity by host cells infiltrating tumour spheroids

K.M. Wilson & E.M. Lord

*Cancer Center and Department of Microbiology and Immunology, University of Rochester, Rochester, NY 14642, USA.*

**Summary** The development of a serum-free, low-protein culture medium has allowed the detection of tumour-specific cytolytic cells in EMT6 immunized mice bearing EMT6 multicellular tumour spheroids. Spheroid associated (SAC) and peritoneal (PC) effector cells were specific to EMT6 as the target cell, not killing line 1, P815 or RIF-1. The natural killer (NK) cell sensitive target YAC-1 was also not lysed by SAC or PC, indicating undetectable levels of NK cells present within infiltrated spheroids. In contrast, high levels of cytolytic activity were present in SAC, PC and spleen cells against WEHI-164, a line sensitive to natural cytotoxic (NC) and macrophage mediated killing. The EMT6 specific acitivity was mediated by Thyl$^+$, Lyt2$^+$ cells. The anti-WEHI-164 effector cell population was Thyl$^-$, Lyt2$^-$. The WEHI-164 killer cells were present in SAC and PC from unimmunized mice while the EMT6 specific effector cells were not. After separation of SAC and PC by size using centrifugal elutriation, anti-EMT6 activity was present only in the lymphocyte fraction while anti-WEHI-164 activity was enriched in the macrophage fraction.

The multicellular tumour spheroid (MTS) (Sutherland *et al.*, 1971) is a useful tumour model because it has many of the three dimensional characteristics of a solid tumour (Sutherland & Durand, 1984; MacDonald & Sordat, 1980). However, unlike solid tumours, MTS are easily dissociated into single cells by a mild trypsinization without loss of cell viability or function. We have used this model previously to demonstrate anti-EMT6 cytolytic activity by spheroid associated cells (SAC) from immunized spheroid bearing mice (Lord, 1980; Lord & Burkhardt, 1984). This anti-EMT6 activity was higher than that of peritoneal (PC) and spleen cells, showing the importance of studying *in situ* host cells (Lord & Burkhardt, 1984). However, since the immunizing EMT6 tumour cells and tumour spheroids were grown in foetal calf serum containing media, it was unclear whether the observed cytotoxicity represented specific anti-tumour immunity.

Cytolytic effector cells can be generated *in vitro* as the result of culture with foetal bovine serum (FBS), or *in vivo* following injection with FBS alone without specific antigen (Golstein *et al.*, 1978). The activation of these cytolytic cells has been attributed to both the antigenic and mitogenic nature of xenogeneic serum. Since FBS components adsorbed or bound onto the surface of cells are not readily removed by washing (Golstein *et al.*, 1978; Kubota, 1984), immunization with tumour cells grown *in vitro* in serum containing media could potentially elicit non-specific effector cells. We have demonstrated the generation of effector cell populations that lyse not only EMT6 but also a number of other tumour cell targets by inoculation of animals with EMT6 cells cultured in FBS supplemented media and/or culture of spleen cells from tumour bearing mice in FBS supplemented media (Reynolds *et al.*, manuscript in preparation). Development of a serum-free, low-protein medium (MAT/P) (Taupier *et al.*, 1985; 1986) in our laboratory for the culture of tumour cells and multicellular tumour spheroids has allowed us to demonstrate specific anti-tumour immunity.

*In situ* antigen specific Thyl$^+$, Lyt2$^+$ cytotoxic T lymphocytes (CTL) can be active in anti-tumour responses (DeLustro & Haskill, 1978). Natural killer (NK) cells and natural cytotoxic (NC) cells have been shown to have spontaneous cytolytic activity against a wide variety of tumours (Lattime *et al.*, 1982). NK cells can be detected in a 4 h chromium release assay and kill NK-sensitive *in vitro* grown tumour lines such as the YAC-1 lymphoma (Lattime *et al.*, 1983). Except for a few animal studies (Ferry *et al.*, 1984), NK activity within tumours has not been observed (Eremin *et al.*, 1981; Totterman *et al.*, 1980). NC activity is against some different targets such as the WEHI-164 sarcoma and is mediated by Thyl$^-$ cells (Lattime *et al.*, 1982; 1983). Recently it has been shown that NC activity may be mediated by tumour necrosis factor (TNF) (Ortaldo *et al.*, 1986). TNF has also been implicated in the lysis of WEHI-164 by mouse resident and peptone-induced peritoneal cells and human fresh, cultured and LPS stimulated monocytes (Colotta *et al.*, 1986; Austgulen *et al.*, 1986; Chen *et al.*, 1985; Kildahl-Anderson *et al.*, 1986; McKinnon, 1986; McKinnon *et al.*, 1986). Since macrophages are present in high numbers in EMT6 solid tumours and MTS (Lord & Keng, 1984; Lord & Burkhardt, 1984), the non-specific cytotoxicity observed in the EMT6 system against WEHI-164 could be mediated by macrophages via TNF.

The objective of this research was to demonstrate specific anti-tumour cytolytic activity by *in situ* host cells in the absence of xenogeneic serum. We examined the specificity of anti-tumour cytolytic activity and characterized the effector cells responsible for anti-EMT6 and anti-WEHI-164 activity.

### Materials and methods

### Mice

BALB/cByJ female mice were purchased from the Jackson Laboratories, Bar Harbor, ME and used at 10–20 weeks of age.

### Tissue Culture Medium

Complete MAT/P (CMP), a serum-free, low-protein medium (Taupier *et al.*, 1985; 1986), was used for all experiments. Proteins used were insulin (0.005 mg ml$^{-1}$) and transferrin (0.005 mg ml$^{-1}$). All media were stored and used under yellow light. Tumour cells lines were adapted to serum-free medium by first reducing FBS concentration from 10% to 2% in MAT/P before using serum-free conditions.

### Tumour system

EMT6 is a mammary sarcoma which arose spontaneously in a BALB/c mouse and was subsequently adapted for tissue culture (Rockwell *et al.*, 1972). The subline designated EMT6/Ro (Rochester) was used for the experiments reported here. All cell lines were tested periodically for mycoplasmal contamination by Mycotrim (Hanna Media Inc., Berkeley, CA). EMT6/Ro was negative for retrovirus and 20 other

Correspondence: K.M. Wilson.
Received 7 April 1986; and in revised form 23 September 1986.

mouse viruses as tested by the MAP test (M.A. Bioproducts, Rockville, MD).

YAC-1, a lymphoma, was induced by Moloney virus in an A/Sn mouse (Cikes *et al.*, 1973). WEHI-164, a fibrosarcoma, was induced by methylcholanthrene in a BALB/c mouse (Rollinghoff & Warner, 1973). RIF-1 (Twentyman *et al.*, 1980), a fibrosarcoma, was induced by radiation in a C3H mouse. P815 (Dunn & Potter, 1957), a mastocytoma, was induced by methylcholanthrene in a DBA/2 mouse. Line-1 (Yuhas *et al.*, 1974), a lung carcinoma, arose spontaneously in a BALB/c mouse.

*Multicellular tumour spheroids*

Multicellular tumour spheroids (MTS) were grown *in vitro* as previously described (Lord *et al.*, 1979). Spheroids were initiated *in vitro* by placing $5\times10^5$ EMT6 tumour cells into 100 mm non-tissue culture petri dishes (Labtek). After 4–5 days the small spheroids were transferred to spinner flasks containing 150 ml MAT/P and medium replenished daily. MTS of 600–800 $\mu$m diameter, obtained after ~2 weeks of growth, were used for all experiments.

*Spheroid implantation and recovery*

MTS (60/mouse) were implanted into the peritoneal cavity using an 18-gauge needle as previously reported (Lord *et al.*, 1979). After 4–6 days spheroids were recovered by flushing the peritoneal cavity with a balanced salt solution (BSS) containing sodium heparin (Gibco) at 5 U ml$^{-1}$. The spheroids were separated from the peritoneal cells by unit gravity sedimentation. Spleens from the same animals were gently teased into single cell suspensions and washed with BSS. Spheroids were washed three times with BSS and dissociated into single cell suspensions with 0.05% trypsin for two 10 min periods at 37°C. Spheroid associated cells (SAC) were washed three times with cold BSS to remove trypsin.

*Immunization*

Mice were immunized 10–14 days prior to spheroid implantation with an i.p. injection of $5\times10^6$ washed, irradiated (50 Gy) EMT6 cells from *in vivo* monolayer culture.

*Cytotoxicity assay*

An 18 h chromium release assay was used to measure cytolytic ability of SAC, PC and spleen cells as previously described (Lord & Burkhardt, 1984*a*; Wilson & Lord, 1986). Two-fold dilutions of effector cells were prepared in 96 well flat bottomed plates. Ten thousand $^{51}$Cr labelled exponentially growing EMT6 target cells were added to each well. The plates were incubated 18 h at 37°C, then 0.1 ml supernatant removed and counted for radioactivity. Spontaneous release was measured by incubating targets with media alone. Complete release was determined by adding a 1:20 dilution of ZAP-isoton (Coulter Electronics) to targets. Four hour assays were identical, with the exception that V bottom plates were used and spun at 200 r.p.m. for 2 min at room temperature after addition of cells. Percent specific lysis (%SL) was calculated from release of radiolabel from target cells, according to the following formula:

$$SL=\frac{\text{(experimental mean–spontaneous mean)}}{\text{(complete mean–spontaneous mean)}}$$

*Morphological analysis*

Slides were prepared using a Shandon–Elliot cytocentrifuge. The slides were air-dried, stained with the Gugol-blue (Wright–Giemsa) method and differential counts made on 300–500 cells per slide.

*Antibody plus complement treatment*

Treatment with cytotoxic monoclonal antibodies (ascites) plus complement was used to remove cell subpopulations prior to chromium release assay. Anti-Thyl.2 was produced by H013.4 (Marshak-Rothstein *et al.*, 1979), and anti-Lyt2.2 by H02.2 (Raulet *et al.*, 1980). Cells at $1\times10^7$ ml$^{-1}$ were suspended in the appropriate dilution (determined by titration) of antibody and placed on ice for 30 min. The cells were then centrifuged, resuspended in the appropriate dilution (determined by titration) of rabbit complement (CedarLane), and incubated at 37°C for 30 min. The cells were then washed, resuspended in medium and counted.

*Centrifugal elutriation*

A centrifugal elutriation procedure was utilized to separate subpopulations of SAC and PC (Lord & Keng, 1984). Single cell suspensions from implanted spheroids were elutriated in media containing 10% newborn calf serum at 4°C. The flow rate was raised from 25 to 41 ml min$^{-1}$ while the rotor speed was decreased in increments from 4000 to 0 r.p.m. and fractions collected at each interval. Lymphocyte fractions had a mean cell volume of 300 $\mu$m while macrophage fractions contained cells ranging from 700–1500 $\mu$m.

### Results

*Cytolytic activity in the absence of foetal bovine serum*

We have previously determined that spheroid associated cells (SAC) recovered from mice immunized with irradiated EMT6 cells and injected with EMT6 multicellular tumour spheroids had cytolytic activity against radiolabelled EMT6 cells (Lord and Burkhardt, 1984). It was a concern that this activity might be the result of bovine serum components adhering to the immunizing cells since xenogeneic serum has been show to elicit cytotoxic cells (Golstein *et al.*, 1978; Reynolds *et al.*, manuscript in preparation). To overcome this potential problem, a serum-free, low-protein defined media (MAT/P) was used for growth of immunizing cells and spheroids, and in the chromium release assay. The added proteins, insulin and transferrin, do not interfere with generation of specific immunity (Reynolds *et al.*, manuscript in preparation). Growth kinetics (size, plating efficiency and number clonogenic cells per spheroid) of tumour spheroids implanted in immunized and unprimed mice using serum-free conditions were similar to those previously published using FBS containing media (Lord & Burkhardt 1984; data not shown).

An 18 h chromium release assay was performed using spheroid associated cells (SAC), peritoneal cells (PC) and spleen cells from immunized spheroid bearing mice as effectors, and monolayer EMT6 as radiolabelled targets. SAC were found to possess the greatest cytolytic ability against EMT6 (Figure 1). The PC also demonstrated cytolytic activity although to a slightly lesser extent. Spleen cells from these mice had little or no cytolytic ability. Thus the host cells infiltrating tumour spheroids were cytolytic in the absence of FBS, and had greater activity than those cells of the periphery.

*Specificity of* in situ *and peritoneal effector cells*

Lysis of EMT6 target cells is observed only in animals immunized with EMT6: no anti-EMT6 activity was detected from SAC when mice were immunized with a different tumour (Lord & Burkhardt, 1984). However, we wished to determine whether the cytolytic effector cells were specific for EMT6 target cells only. To determine the target specificity of SAC and PC, a panel of radiolabelled targets was used in a chromium release assay. RIF-1 (a C3H radiation induced fibrosarcoma) and line 1 (a BALB/c lung carcinoma) were not lysed by SAC and PC effector cells (Table

**Figure 1** Comparison of *in situ* and peripheral cytolytic activity in the absence of foetal bovine serum. 18 h chromium release assay of SAC (△), PC (□) and spleen cells (●) from immunized MTS bearing mice against EMT6 target cells. The difference between SAC and PC cytolytic activity was significant at the 95% confidence interval by lytic units analysis.

**Figure 2** Titration curve of anti-WEHI-164 cytolytic activity. 18 h chromium release assay of SAC (△), PC(□) and spleen cells (●) against WEHI-164 targets.

**Table I** Target lysis specificity of SAC, PC: % specific lysis at various effector to target ratios

| | *SAC* | | | *PC* | | |
|---|---|---|---|---|---|---|
| *Target* | *35:1* | *18:1* | *9:1* | *100:1* | *50:1* | *25:1* |
| *Experiment 1 – 18 hour chromium release assay* | | | | | | |
| EMT6 | 51.1 | 52.0 | 38.8 | 51.5 | 34.6 | 24.5 |
| Line-1 | 0 | 0 | 6.2 | 3.2 | 0 | 0 |
| P815 | 39.1 | 7.1 | 11.9 | 28.1 | 2.8 | 0 |
| *Experiment 2 – 18 hour chromium release assay* | | | | | | |
| | *50:1* | *25:1* | *12:1* | | | |
| EMT6 | 24.4 | 21.6 | 10.9 | 23.0 | 18.9 | 9.1 |
| RIF-1 | 1.5 | 5.8 | 6.6 | 9.8 | 3.0 | 3.6 |
| *Experiment 3a – 18 hour chromium release assay* | | | | | | |
| EMT6 | 66.8 | 54.9 | 43.5 | 61.4 | 53.5 | 36.0 |
| WEHI-164 | 17.3 | 20.0 | 31.8 | 35.1 | 44.4 | 36.6 |
| *Experiment 3b – 4 hour chromium release assay* | | | | | | |
| EMT6 | 18.8 | 11.7 | 6.4 | 16.3 | 9.3 | 3.9 |
| WEHI-164 | 4.2 | 4.3 | 3.1 | 7.6 | 4.3 | 2.5 |
| YAC-1 | 3.9 | 3.0 | 2.5 | 19.4 | 16.3 | 10.5 |

I). P815 (a DBA/2 mastocytoma) was not killed except at the highest effector to target ratio which may have been influenced by crowded culture conditions. YAC-1, an A/Sn lymphoma and NK sensitive line, was not killed by SAC in a 4 h chromium release assay although some lysis by PC was present. A 4 h assay was used for this target since non-specific lysis by radiolabelled YAC-1 cells was too great at 18 h to yield meaningful data. WEHI-164, an NC and macrophage but not NK sensitive line (Lattime *et al.*, 1982; 1983; Chen *et al.*, 1985; Ortaldo *et al.*, 1986) was killed by both the SAC and PC effector cells.

### *Characterization of the specific (anti-EMT6) and non-specific (anti-WEHI-164) cytolytic activity*

The anti-WEHI-164 killing was very high, especially by SAC and PC from immunized mice (Figure 2). This killing was consistently higher than the anti-EMT6 activity at the lower effector to target ratios (Figures 1 and 2).

The anti-WEHI-164 but not the anti-EMT6 activity was present in spleens from spheroid bearing mice (Figures 1 and 2). The anti-WEHI effector cell was also demonstrable in SAC and PC from unprimed mice while the anti-EMT6 cell was not (Figure 3).

**Figure 3** Comparison of cytolytic activity against EMT6 and WEHI-164 in unimmunized spheroid bearing mice. 18 h chromium release assay of SAC (●) and PC (□) *vs.* EMT6 (A) and WEHI-164 (B).

It was of interest to determine whether the anti-EMT6 and anti-WEHI-164 activities were carried out by the same or different cell populations. To determine the phenotype of the anti-EMT6 and anti-WEHI-164 effector cells, SAC and PC were treated with either anti-Thyl or Lyt2 plus complement prior to the chromium release assay. Antibody plus complement treatment removed the anti-EMT6 activity. Thus $Thyl^+$, $Lyt2^+$ cells were responsible for the anti-EMT6 activity (Figure 4). Antibody plus complement treatment did not remove anti-WEHI-164 activity; therefore, the anti-WEHI-164 effector cells were $Thyl^-$, $Lyt2^-$ (Figure 4). These data indicate that the EMT6 and WEHI-164 killers are different cell populations.

To further characterize the cells responsible for killing EMT6 and WEHI-164, centrifugal elutriation was used to

**Figure 4** Characterization of effector cell responsible for cytolytic activity. 18 h chromium release assay of SAC (A, B) and PC (C, D) against EMT6 (A, C) and WEHI-164 (B, D) targets. Cells were treated with media alone (●), complement alone (□), anti-Thyl + complement (○) or anti-Lyt2 + complement (△) immediately prior to assay.

**Table II** Differential counts from elutriated fractions

| *Fraction* | *% Lymphocytes* | *% Macrophages* | *% Granulocytes* | *% Tumour cells* |
|---|---|---|---|---|
| SAC unsep. | 9.2 ± 0.5[a] | 34.0 ± 7.2 | 8.8 ± 0.5 | 48.0 ± 6.7 |
| SACL | 56.6 ± 7.2 | 14.4 ± 1.6 | 29.0 ± 8.8 | 0 |
| SACM | 2.5 ± 1.5 | 70.4 ± 2.9 | 0.8 ± 0.2 | 26.3 ± 4.2 |
| PC unsep. | 57.1 ± 4.9 | 34.7 ± 3.8 | 8.2 ± 1.0 | 0 |
| PCL | 82.5 ± 10.0 | 6.4 ± 1.0 | 11.0 ± 11.0 | 0 |
| PCM | 15.7 ± 6.3 | 79.6 ± 6.0 | 4.7 ± 0.4 | 0 |

[a]SEM.

separate SAC and PC subpopulations based on size. Differential counts of the lymphocyte and macrophage fractions (Table II) indicated a 5 to 20 fold enrichment for one cell type over the other. Anti-EMT6 activity was present exclusively in the lymphocyte fraction (Figure 5). Anti-WEHI-164 activity was present in each fraction but greatest in the macrophage fraction (Figure 5).

### Discussion

The multicellular tumour spheroid (MTS) is useful as a tumour model due to its tumour-like 3-dimensional structure and ease in manipulation (Sutherland *et al.*, 1971; MacDonald & Sordat, 1980; Sutherland & Durand, 1984). This model is especially useful for examining the host cells which infiltrate tumours. These *in situ* host immune cells (macrophages, granulocytes, lymphocytes) are often present in different proportions and activity levels than those cells of the periphery (Lord, 1980; Lord & Burkhardt, 1984). The MTS model was used to characterize the infiltrating host cell population responsible for cytolytic activity against tumour cells and the target lysis specificity.

It was a concern that the cytolytic activity of SAC demonstrated in previous studies might be due to foetal bovine serum used to culture cells used in immunization and in growing MTS. To rule out this possibility, a serum-free, low-protein medium was developed in our laboratory (Taupier *et al.*, 1985; 1986) and used exclusively in these experiments. Cytolytic activity was present in infiltrated EMT6 spheroids from immunized syngeneic BALB/c mice (Figure 1). This activity was greater than that of peritoneal cells (PC) while spleen cells had very little activity.

EMT6 spheroids implanted into immunized syngeneic mice become infiltrated by macrophages, granulocytes and lymphocytes (Lord & Burkhardt, 1984; Table II). Removal of either Thyl$^{+}$ or Lyt2$^{+}$ cells abrogated activity (Figure 4). Thus the cell responsible for anti-EMT6 cytotoxicity was a Thyl$^{+}$, Lyt2$^{+}$ cytotoxic T lymphocyte.

The target cell specificity of SAC and PC was determined by utilizing a panel of radiolabelled target cells in the chromium release assay. The SAC effector cells failed to kill several tumour lines including YAC-1, an NK-sensitive line (Table I). This is in agreement with some others who have been unable to demonstrate NK cells within tumours (Eremin *et al.*, 1981; Totterman *et al.*, 1980). It was

**Figure 5** Separation of effector cells by centrifugal elutriation. 18 h chromium release assay of SAC (A, B) and PC (C, D) against EMT6 (A, C) and WEHI-164 (B, D) targets. Cells were separated by centrifugal elutriation into lymphocyte (○) and macrophage (●) enriched fractions prior to assay.

surprising, however, that the SAC effector cells killed WEHI-164 to an even greater extent than EMT6 (Figures 1 and 2). These anti-WEHI-164 killers (Thyl$^-$, Lyt2$^-$) were a different population of cells than the anti-EMT6 effectors (Thyl$^+$, Lyt2$^+$) (Figure 4). This non-specific activity against WEHI-164 was also present in host infiltrated tumour spheroids of line 1, a progressive BALB/c lung carcinoma (data not shown). The anti-WEHI-164 killers were present in spleen cells, SAC and PC from unimmunized mice where anti-EMT6 activity was not evident (Figure 3).

The anti-WEHI-164 activity may have been mediated by NC cells (Lattime *et al.,* 1982; 1983). Recently it has been shown that NC activity may be mediated by TNF, a macrophage derived factor (Ortaldo *et al.,* 1986). Since macrophages are present in high numbers *in situ* in the EMT6 system (Lord & Burkhardt, 1984; Lord & Keng, 1984; Table II) it is possible that this activity was mediated by a macrophage secreted cytotoxin such as TNF (Kildahl-Anderson, *et al.,* 1986; Chen *et al.,* 1985; Colotta *et al.,* 1986; Austgulen *et al.,* 1986). In support of this hypothesis we found that anti-WEHI-164 activity was greater in fractions of effector cells enriched for macrophages by centrifugal elutriation than in fractions enriched for lymphocytes (Figure 5). These results indicate that while a lymphocyte population is responsible for the specific anti-EMT6 activity, the anti-WEHI-164 activity may be mediated by macrophages. This activity is present at very low effector to target ratios and is very high in the *in situ* host cell population. Preliminary data suggest that this activity is carried out by a secreted factor (data not shown).

It is interesting that while anti-EMT6 activity was exclusively in the lymphocyte fraction, anti-WEHI-164 activity was present to some extent in all fractions although it was highest in the macrophage fraction. This may be due to factor production by the small number of contaminating macrophages over the 18 h assay period, since the activity seen is high even at very low effector to target ratios. It is also interesting that EMT6 grows progressively as a solid tumour or tumour spheroids in unimmunized mice (data not shown). This progressive tumour growth occurs despite the presence of macrophages capable of cytolytic activity. These results indicate that EMT6 is insensitive to this macrophage mediated killing, but does not rule out possible cytostatic effects.

We have demonstrated direct cytotoxicity of EMT6 by host cells recovered from MTS in a serum-free system. These anti-EMT6 effectors cells were Thyl$^+$, Lyt2$^+$ and specific for EMT6 targets. Thyl$^-$, Lyt2$^-$ cells, most likely macrophages, which mediated WEHI-164 killing were also present in SAC and PC from unimmunized as well as immunized spheroid bearing mice. Additional experiments are in progress to further characterize the WEHI-164 killing and its significance in anti-tumour immunity.

This work was supported by Grant CA 28332 from the National Cancer Institute of the National Institutes of Health. We thank Dr. Peter Keng for critical review of the manuscript and Robert Wilson for computer software development.

### References

AUSTGULEN, R., HAMMERSTROM, J., ESPEVIK, T. & NISSEN-MEYER, J. (1986). Human monocyte cytotoxic factor mediates cytolysis of WEHI 164 cells. *Cell. Immunol.*, **98**, 211.

CHEN, A.R., McKINNON, K.P. & KOREN, H.S. (1985). Lipopolysaccharide (LPS) stimulates fresh human monocytes to lyse Actinomycin D-treated WEHI-164 target cells via increased secretion of a monokine similar to tumor necrosis factor. *J. Immunol.*, **135**, 3978.

CIKES, M., FRIBERG, S. & KLEIN, G. (1973). Progressive loss of H-2 antigens with concomitant increase of cell-surface antigen(s) determined by moloney leukemia virus in cultured murine lymphomas. *J. Natl Cancer Inst.*, **50**, 347.

COLOTTA, F., POLENTARUTTI, N., BERSANI, L., POLI, G. & MANTOVANI, A. (1986). Rapid killing of Actinomycin D-treated tumor cells by mononuclear phagocytes: Characterization of effector cells in mice. *J. Leuk. Biol.*, **39**, 205.

DELUSTRO, F. & HASKILL, J.S. (1978). *In situ* cytotoxic T-cells in a methylcholanthrene induced tumor. *J. Immunol.*, **121**, 1007.

DUNN, T.B. & POTTER, M. (1957). A transplantable mast-cell neoplasm in the mouse. *J. Natl Cancer Inst.*, **18**, 587.

EREMIN, O., COOMBS, R.R.A. & ASHBY, J. (1981). Lymphocytes infiltrating human breast cancers lack K-cell activity and show low levels of NK-cell activity. *Br. J. Cancer*, **44**, 166.

FERRY, B.L., FLANNERY, G.R., ROBINS, R.A., LAWRY, J. & BALDWIN, R.W. (1984). Phenotype of cytotoxic effector cells infiltrating a transplanted chemically induced rat sarcoma. *Immunol.*, **53**, 243.

GOLSTEIN, P., LUCIANI, M.F., WAGNER, H. & ROLLINGHOFF, M. (1978). Mouse T-cell-mediated cytolysis specifically triggered by cytophilic xenogeneic serum determinants: A caveat for the interpretation of experiments done under 'syngeneic' conditions. *J. Immunol.*, **121**, 2533.

KILDAHL-ANDERSON, O. AUSTGULEN, R. & NISSEN-MEYER, J. (1986). WEHI 164 sarcoma cells rendered resistant to monocyte-released cytotoxin are also resistant to monocyte-induced cytolysis. *Cancer Immunol. Immunother.*, **21**, 77.

KUBOTA, K. (1984). Association of serum beta-2-microglobulin with H-2 class I heavy chains on the surface of mouse cells in culture. *J. Immunol.*, **133**, 3203.

LATTIME, E.C., PECORARO, G. & STUTMAN, O. (1982). Natural cytotoxic cells against solid tumors in mice. IV. Natural cytotoxic (NC) cells are not activated natural killer (NK) cells. *Int. J. Cancer*, **30**, 471.

LATTIME, E.C., PECORARO, G.A., CUTTITO, M.J. & STUTMAN, O. (1983). Murine non-lymphoid tumors are lysed by a combination of NK and NC cells. *Int. J. Cancer*, **32**, 523.

LORD, E.M., PENNY, D.P., SUTHERLAND, R.M. & COOPER, R.A. (1979). Morphological and functional characteristics of cells infiltrating and destroying tumor multicellular spheroids *in vivo*. *Virchows Arch. B Cell Pathol.*, **31**, 103.

LORD, E.M. (1980). Comparison of *in situ* and peripheral host immunity to syngeneic tumours employing the multicellular spheroid model. *Br. J. Cancer*, **41**, 123.

LORD, E.M. & BURKHARDT, G. (1984). Assessment of *in situ* host immunity to syngeneic tumors utilizing the multicellular spheroid model. *Cell. Immunol.*, **85**, 340.

LORD, E.M. & KENG, P.C. (1984). Methods for using centrifugal elutriation to separate malignant and lymphoid cell populations. *J. Immunol. Meths.*, **68**, 147.

MACDONALD, H.R. & SORDAT, B. (1980). The multicellular tumor spheroid: A quantitative model for studies of *in situ* immunity. *Contemp. Top. Immunobiol.*, **10**, 317.

MARSHAK-ROTHSTEIN, A., FINK, P. GRIDLEY, T., RAULET, D.H., BEVAN, M.J. & GEFTER, M.L. (1979). Properties and applications of monoclonal antibodies directed against determinants of the Thy-1 locus. *J. Immunol.*, **122**, 2941.

MCKINNON, K.P., CHEN, A.R., ARGOV, S., LANE, B.C. & KOREN, H.S. (1986). Cytolysis of Actinomycin D-treated target cells by cell-free supernatants from human monocytes. *Immunobiol.*, **171**, 27.

ORTALDO, J.R., MASON, L.H., MATHIESON, B.J., LIANG, S.M., FLICK, D.A. & HERBERMAN, R.B. (1986). Mediation of mouse natural cytotoxic activity by tumour necrosis factor. *Nature*, **321**, 700.

RAULET, D.H., GOTTLEIB, P.D. & BEVAN, M.J. (1980). Fractionation of lymphocyte populations with monoclonal antibodies specific for Lyt2.2 and Lyt3.1. *J. Immunol.*, **125**, 1136.

ROCKWELL, S.C., KALLMAN, R.F. & FAJARDO, L.F. (1972). Characteristics of a serially transplanted mouse mammary tumor and its tissue culture adapted derivative. *J. Natl Cancer Inst.*, **49**, 735.

ROLLINGHOFF, M. & WARNER, N.L. (1973). Specificity of *in vivo* tumor rejection assessed by mixing spleen cells with target and unrelated tumor cells. *Proc. Soc. Exp. Biol. Med.*, **144**, 813.

SUTHERLAND, R.M., MCCREDIE, J.A. & INCH, W.R. (1971). Growth of multicell spheroids in tissue culture as a model of nodular carcinomas. *J. Natl Cancer Inst.*, **46**, 113.

SUTHERLAND, R.M. & DURAND, R.E. (1984). Growth and cellular characteristics of multicell spheroids. In *Recent Results in Cancer Research: Spheroids in Cancer Research,* Acker, H. *et al.* (eds) p. 24. Springer-Verlag: Berlin.

TAUPIER, M.A., REYNOLDS, S.D. & LORD, E.M. (1985). Culture of tumor cells in serum free low protein media allows detection of antigens previously masked. *Fed. Proc.*, **44**, 961.

TAUPIER, M.A., REYNOLDS, S.D., BIDLACK, J.M. & LORD, E.M. (1986). Development of a serum-free very low protein medium allowing long-term culture of a wide variety of tumor cells. (submitted).

TOTTERMAN, T.H., PARTHENAIS, E., HAYRY, P., TIMONEN, T. & SAKSELA, E. (1980). Cytological and functional analysis of inflammatory infiltrates in human malignant tumor. III. Further functional investigations using cultured autochthonous tumor cell lines and freeze-thawed infiltrating inflammatory cells. *Cell. Immunol.*, **55**, 219.

TWENTYMAN, P.R., BROWN, J.M., GRAY, J.W., FRANKO, A.J., SCOLES, M.A. & KALLMAN, R.F. (1980). A new mouse tumor model system (RIF-1) for comparison of endpoint studies. *J. Natl Cancer Inst.*, **64**, 595.

WILSON, K.M. & LORD, E.M. (1986). Effects of radiation on host-tumor interactions using the multicellular tumor spheroid model. *Cancer, Immunol. Immunother.*, **23**, 20.

YUHAS, J.M., PAZMINO, N.H., PROCTOR, J.O. & TOYA, R.E. (1974). A direct relationship between immune competence and the subcutaneous growth of a malignant murine lung tumor. *Cancer Res.*, **34**, 722.